# TGF-β in fibrosis by acting as a conductor for contractile properties of myofibroblasts

**DOI:** 10.1186/s13578-019-0362-3

**Published:** 2019-12-09

**Authors:** Alexandre Vallée, Yves Lecarpentier

**Affiliations:** 1 Délégation à la Recherche Clinique (DRCI), Hôpital Foch, Suresnes, France; 2 DACTIM-MIS, Laboratoire de Mathématiques et Applications (LMA), CNRS, UMR 7348, Université de Poitiers, CHU de Poitiers, Poitiers, France; 3 Centre de Recherche Clinique, Grand Hôpital de l’Est Francilien (GHEF), Meaux, France

**Keywords:** Transforming growth factor-β1, PPARγ, Canonical WNT/-β-catenin, YAP/TAZ, Smad, Myofibroblasts, Fibrosis, Myosin

## Abstract

Myofibroblasts are non-muscle contractile cells that play a key physiologically role in organs such as the stem villi of the human placenta during physiological pregnancy. They are able to contract and relax in response to changes in the volume of the intervillous chamber. Myofibroblasts have also been observed in several diseases and are involved in wound healing and the fibrotic processes affecting several organs, such as the liver, lungs, kidneys and heart. During the fibrotic process, tissue retraction rather than contraction is correlated with collagen synthesis in the extracellular matrix, leading to irreversible fibrosis and, finally, apoptosis of myofibroblasts. The molecular motor of myofibroblasts is the non-muscle type IIA and B myosin (NMMIIA and NMMIIB). Fibroblast differentiation into myofibroblasts is largely governed by the transforming growth factor-β1 (TGF-β1). This system controls the canonical WNT/β-catenin pathway in a positive manner, and PPARγ in a negative manner. The WNT/β-catenin pathway promotes fibrosis, while PPARγ prevents it. This review focuses on the contractile properties of myofibroblasts and the conductor, TGF-β1, which together control the opposing interplay between PPARγ and the canonical WNT/β-catenin pathway.

## Background

Gabbiani et al. first reported the presence of modified fibroblasts or myofibroblasts in granulation tissue and its role in wound healing [1]. They showed that the modified fibroblasts presented contractile properties, shared some similarities with smooth muscle cells, and played a major role in wound contraction. In fact, it was observed as early as 1916 that the contraction-retraction process is an important factor in the repair of tissue injury [2]. The active retraction of granulation tissue in a wound is due to non-contractile muscle cells known as myofibroblasts [3]. Transforming growth factor-β1 (TGF-β1) acts on the dual antagonism system comprising the canonical WNT/β-catenin and peroxisome proliferator activated receptor gamma (PPARγ). These two signaling systems act in an opposite manner in several pathological conditions [4–12]. Thus, when the expression of PPARγ is increased, WNT/β-catenin signaling is decreased, while when WNT/β-catenin signaling is increased, PPARγ expression is decreased [11, 13]. Myofibroblast differentiation is controlled by TGF-β1, which activates canonical WNT signaling and decreases PPARγ expression [14].

Fibroblasts and myofibroblasts are the main effectors involved in the initiation of fibrosis due to excessive collagen deposition and an inappropriate extracellular matrix (ECM). TGF-β1 activation leads to the differentiation of fibroblasts into contractile myofibroblasts, expression of α-smooth muscle actin (α-SMA) and the synthesis of proteins of the extracellular matrix such as collagen. This review focuses on homeostasis and the contractile properties of myofibroblasts in physiological and pathophysiological states, mainly in the fibrosis process. The study is mainly based on the role of the opposite conductor TGFβ1 on both PPARγ and the canonical WNT/β-catenin pathway.

## Myofibroblasts

According to the first descriptions, the major structural characterization of contractile myofibroblasts is the presence of actin filament bundles containing α-SMA, peripheral focal adhesions and adjoining myofibroblast junctions composing the granulation tissue [1, 15]. Soon after injury, local fibroblasts called proto-myofibroblasts translate into the core of the wound. Thus, proto-myofibroblasts differentiate into myofibroblasts containing α-SMA, leading to wound retraction [16]. Proto-myofibroblasts contain type I and III collagen and fibronectin EDA, which is indispensable for the differentiation of myofibroblasts [17]. After closing the wound, myofibroblasts disappear by apoptosis [18].

The origins of myofibroblasts are multiple [19]. Mesenchymal stem cells (MSCs) and fibroblasts, which are the precursors of proto-differentiated myofibroblasts, have been observed in physiological tissues such as the alveolar septa of the lung, uterine submucosa, lymph nodes, spleen, the renal capsule, the periodontal ligament, intestinal crypts and stromal bone marrow. In general, fibroblast differentiation into myofibroblasts occurs during the skin repair process following injury, during the fibrotic process in the liver, skin, kidneys, heart and lungs and in the stroma of cancers. In the granulation tissue, myofibroblasts induce remodeling of the ECM and then promote wound healing [20]. However, the aberrant wound healing sometimes leads to a proliferation of myofibroblasts. Their differentiation can be triggered by multiple cell pathways [21]. Growth factors are usually carried in the ECM. They are stimulated and then released by mechanical stress or proteolytic cleavage. Then, they migrate to bind to membrane receptors. This phenomenon leads to activation of the intracellular complexes that migrate to the nucleus, thus promoting the transcription or repression of target genes that are involved in fibrosis.

Myofibroblasts have been observed in several fibrosis processes, including systemic sclerosis (SSc), glomerulosclerosis, idiopathic pulmonary fibrosis, liver cirrhosis, heart failure and myocardial infarction [22]. Myofibroblasts have also been observed in the stroma in epithelial cancers [23], retinal detachments [24] and human capsular cataracts [25]. Chronic injury causes prolonged stimulation of fibroblasts [26] for the differentiation into myofibroblasts. Myofibroblasts could remain after the closure of a wound, which results in a hypertrophic scar, especially after burns [27]. Myofibroblasts may be found in coronary plaques [28]. Precursors of myofibroblasts are stellate cells in the liver [29], pericytes in the kidneys [30] and fibrocytes derived from bone marrow [31]. Lines of non-fibroblast cells [32, 33] can differentiate into myofibroblasts through the epithelial-mesenchymal transition [34] and the endothelial-mesenchymal transition [35]. MSCs are precursors of myofibroblasts in several diseases [36]. A major case is the presence of myofibroblasts in physiological human placenta, a tissue in which contractile myofibroblasts constitute the majority of the placental stem villi [37]. In normal human placenta, the differentiation of fibroblasts into myofibroblasts occurs from the peripheral part of the stem villi towards its main part. Fibroblast differentiation into myofibroblasts requires the participation of complex chemical and physical factors. Among these are the augmentation index of stiffness of the studied tissues [16] and TGF-β1 with fibronectin EDA [17]. TGF-β1 increases α-SMA synthesis leading to myofibroblast differentiation. Incorporation of α-SMA in stress fibers stimulates the contractile properties of myofibroblasts [38]. ECM enables the transmission of the contractile force, enhanced by α-SMA and the myosin molecular motor through focal adhesions containing transmembrane integrins [39]. In ECM, TGF-β1 is released by a mechanical, integrin-dependent process [27]. Moreover, the availability of TGF-β1 is stimulated by the rigidity of the ECM [40].

## Myofibroblasts are contractile cells

There are two types of contractile cells, i.e. contractile muscle cells and non-muscle cells. All the contractile cells act by means of a myosin molecular motor, which is associated with α-SMA. The molecular motors are type I and type II muscle myosins (MMI and II) in muscle cells (smooth muscles and striated sarcomeric muscles) and type II non-muscle myosins (NMMII) in non-muscle contractile cells [41]. NMMII is involved in cell polarity generation, cell migration and cell–cell adhesion. The molecular motor is the non-muscle myosin IIA and B (NMMIIA and B). NMMIIA is largely dominant in myofibroblasts and predominates in the extravascular part of normal human placental stem villi [42]. Myofibroblasts are also observed in numerous pathophysiological tissues, including cancer (breast carcinoma, epithelial cells of cancerous mammary glands) and in fibrotic processes (Dupuytren nodules, hypertrophic scars) [43].

As with MMII, NMMII contains three pairs of chains: one pair of heavy chains and two pairs of light chains that regulate and stabilize the heavy chain structure. Two main systems regulate NMMII activity: first, the calcium-calmodulin-myosin light chain kinase (MLCK); secondly, the Rho/ROCK/myosin light chain phosphatase [44].

**Fig. 1:**
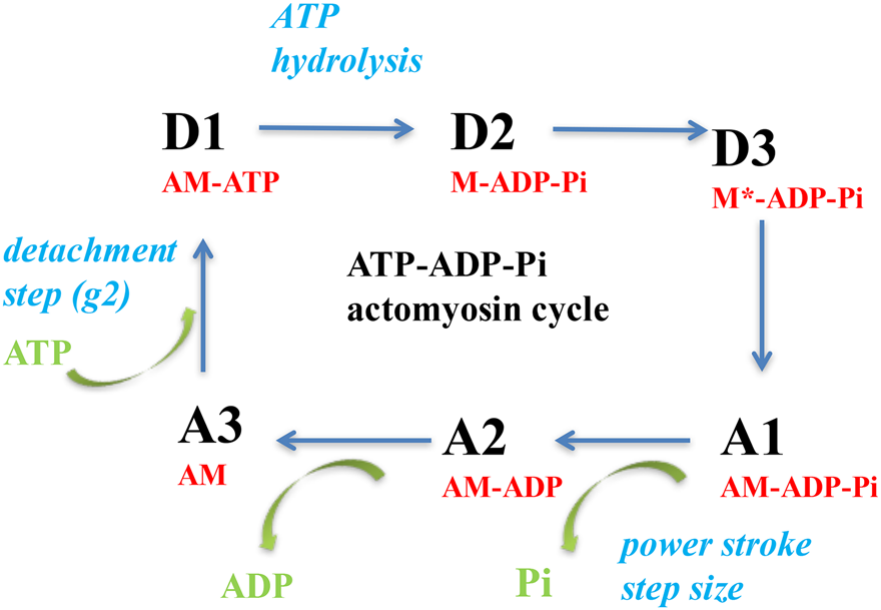
Actin-myosin crossbridge (CB) cycle [57]. Cycle of ATP-ADP-Pi actin-myosin interaction. The CB cycle is composed of six successive conformational steps, i.e. three detached steps (D1, D2, and D3) and three attached steps (A1, A2, and A3). Transition A**3 →** D1 is the ATP binding step that induces CB detachment after ATP binding to the actin (A)-myosin (M) complex (AM). The rate constant for detachment is g_2_: AM **→ **A + M. Transition D1 **→** D2 is the ATP hydrolysis: M + ATP **→** M-ADP-Pi. Transition D2 **→** D3 is M-ADP-Pi **→** M*-ADP-Pi. D3 is the step with the highest probability. Transition D3 **→ **A1is the attachment state: the myosin head (M*-ADP-Pi) binds to A and the rate constant for attachment is f_1_: M-ADP-Pi + A **→** AM-ADP-Pi. Transition A1 **→ **A2 is the power stroke which is triggered by the Pi release: AM-ADP-Pi **→** AM-ADP + Pi. The power stroke is characterized by the generation of a unitary CB force ($\cong$ picoN) and an elementary CB step ($\cong$ nm). Transition A2 **→** A3 is the release of ADP: AM-ADP **→** AM + ADP

NMMIIA binds to actin in the head of the heavy chain. Myosin filaments link actin filaments and the NMMIIA molecules assemble into bipolar filaments [41]. This allows the myosin to slide along the actin filaments. A bend in the head also enables a conformational change in the actin filaments so that they are no longer operating in a parallel manner, but are opposed to one another. The crossbridge (CB) actin-myosin cycle is very similar to that observed in the myosin of smooth and striated muscles. An ATP molecule binds with the NMMIIA-ATPase site on the myosin head. This enables the dissociation of actin from the NMMIIA head. Then, the ATP is hydrolyzed and the NMMIIA is able to bind with actin. Thus, the binding process results in a bend in the NMMIIA head, leading to the creation of a single CB force (order of magnitude: few picoNewtons) and a displacement of a few nanometers. The actin-NMMIIA complex then releases ADP. A new ATP molecule dissociates actin from the NMMIIA head, and a new CB cycle can begin (Fig. 1).

The main feature of NMMIIA is its extreme slowness. NMMIIA kinetics are extremely slow (Table 1) [45]. Compared to skeletal or smooth muscles, the constants of CB detachment and CB attachment, the catalytic constant, and myosin ATPase are low (Table 1). Nevertheless, the single force of one NMMIIA CB is of the same order of magnitude as that observed in smooth and striated muscles. The low isometric tension reported in placental stem villi [46] is explained by the low placental myosin content [47]. The extremely slow shortening velocity is explained by the low constant of detachment [45, 47]. From a thermodynamic standpoint, force and flow, and the rate of entropy production, are particularly low compared to that observed in striated muscles [48].

**Table 1:** Comparative molecular properties of non-muscle myosin (NMII) and muscle myosin (MII)

	Placenta	Trachea	Soleus	EDL	Heart
π	2.1 (0.3)	1.9 (0.1)	2.3 (0.1)	2.1 (0.1)	1.6 (0.1)
f_1_	0.07 (0.05)	8.9 (2.4)	38 (11)	242 (54)	306 (89)
g_2_	0.33 (0.24)	33.5 (8.4)	328 (99)	1162 (199)	730 (15)
ATPase activity	4.8 (0.1)	2.9 (1.1)	5 (2)	50 (22)	283 (177)

Four main parameters of crossbridge (CB) myosin molecular motors are presented. CB unitary force (π; in pN); constant of CB attachment (f_1_; in s^−1^); constant of CB detachment (g_2_; in s^−1^); maximum ATPase activity (in nM g^−1^ s^−1^). Values ± SD were determined in human placenta [40], and in rat trachea, soleus, EDL and heart [50]. CB kinetics were dramatically low in non-muscle placental myofibroblasts compared with values reported in myocytes of trachea, soleus, EDL and heart. Only, CB unitary force was of same order of magnitude in both myofibroblasts and muscle cells

In myofibroblasts from human placenta, the mechanical behavior of the actin-myosin apparatus is similar to that of smooth and striated muscles. The contraction phase is enhanced by either an electric field or addition of KCl. The relaxation is increased by either 2,3-butanedione monoxime (BDM), an inhibitor of NMMII, or isosorbide dinitrate (ISDN), a NO donor [46].

In addition to the ability of myofibroblasts to contract and relax like myocytes in muscles, they share other fundamental properties, namely with respect to the Frank Starling phenomenon and the A.V. Hill hyperbolic relationship.

The Frank Starling phenomenon [49] is based on the link between the initial length of myocardial fibers and the force generated by contraction. There is a predictable association between the length between sarcomeres and the tension of the muscle fibers. There is a particular length between sarcomeres at which the tension in muscle fibers is greatest, leading to the greatest contraction force. The Franck Starling phenomenon is the observation that ventricular output increases as preload (end-diastolic pressure) increases [50, 51].

The left ventricular performance curves relate preload, measured as the left ventricular end-diastolic volume or pressure, to cardiac performance, measured as ventricular stroke volume or cardiac output. On the curve of a normally functioning heart, cardiac performance increases continuously as preload increases. During states of increased left ventricular contractility, there is a greater cardiac performance for a given preload. This is represented graphically as an upward shift of the normal curve. Conversely, during states of decreased left ventricular contractility associated with systolic heart failure, there is less cardiac performance for a given preload as compared to the normal curve. This is represented by a downward shift of the normal curve. Decreased contractility can also result from a loss of myocardium, as is also observed with myocardial infarction, β-blockers (acutely), non-dihydropyridine Ca^2+^ channel blockers, and dilated cardiomyopathy [52].

Changes in afterload, which is the resistance force that the ventricle must overcome to empty contents at the beginning of systole, will also shift the Frank-Starling curve. A decrease in afterload will cause an upward shift of the ventricular performance curve in a similar fashion to an increase in inotropy. Conversely, an increase in afterload will cause a downward shift of the curve in a similar fashion to a decrease in inotropy.

An increase in catecholamines, such as norepinephrine, during exercise will result in an upward shift of the Frank-Starling curve. Catecholamines achieve this increase by binding to a myocyte β1-adrenergic receptor, a g-protein coupled receptor, ultimately resulting in an increased Ca^2+^ channel release from the sarcoplasmic reticulum which enhances the force of contraction [53].

The Frank–Starling mechanism plays a role in the compensation of systolic heart failure, buffering the fall in cardiac output to help preserve sufficient blood pressure to perfuse the vital organs. Heart failure caused by the impaired contractile function of the left ventricle causes a downward shift of the left ventricular performance curve. At any given preload, the stroke volume will be decreased with respect to normal. This reduced stroke volume leads to incomplete left ventricular emptying. Consequently, the volume of blood that accumulates in the left ventricle during diastole is greater than normal. The amplified residual volume increases the stretch of the myocardial fibers and induces a greater stroke volume with the next contraction, via the Frank-Starling mechanism. This allows for better emptying of the enlarged left ventricle and preserves cardiac output [54].

This phenomenon expresses the fact that the force developed by striated and smooth muscles depends on their initial length, which in turn depends on their preload. The longer the initial length (or the greater the preload), the greater the active tension (AT) (the active tension is the difference between total tension (TT) and preload or resting tension (RT) (AT = TT − RT).

This phenomenon has been reported in the human placenta. Thus, modifications in the volume of the intervillous chamber modify the length of the placental stem villi. Through the Frank Starling phenomenon, contraction of myofibroblasts in placental stem villi modulates the distal resistances of the umbilical artery [46].

Secondly, myofibroblastic tissues present the hyperbolic relationship of A.V. Hill, between the maximum shortening velocity and the level of isotonic tension [55]. This mechanical property is fundamental because it allows the formalism of A. Huxley to be applied to this type of contractile tissue [56].

Huxley’s equations [56] describe the behavior of cross-bridge molecular motors from a phenomenological point of view in both contractile muscle and non-muscle tissues. The considerable number of cross-bridges involved in muscle and non-muscle contractile systems provides the necessary grounds for using statistical mechanics. The probabilities of several elementary steps in the actin–myosin cycle in contractile structures can thus be calculated. The combination of statistical mechanics [57] with Huxley’s formalism [56] makes it possible to calculate numerous thermodynamic parameters of the system under study.

The Huxley formalism states that the curvature (G) of the hyperbola is related to the thermodynamic properties of the muscular system (the tension-velocity relationship of the contractile structures). Moreover, it is important to know the asymptotes and the curvature G of the hyperbolic relationship that must be introduced into the Huxley equations. These equations are fundamental because they allow us to calculate:

- The number of CBs per unit volume of contractile tissue;
- The average unitary force of an actin-myosin interaction.

The attachment constant (fl) and detachment constants (g1 and g2) of actin from myosin CBs:

- The molar concentration of NMMII.
- The catalytic constant (kcat), which is the inverse of the duration of an actin-myosin cycle.
- The maximum ATPase activity of NMMII which is the product of kcat and the molar concentration of NMMII.
- The efficiency of the actin-myosin interaction.

In addition, Huxley’s formalism leads to the calculation of the probability of the different attachment and detachment steps that the NMMII molecule undergoes during the cycle.

In our laboratory, we have developed the formalism of statistical mechanics using the grand canonical ensemble for the calculation of many thermodynamic factors, including the statistical entropy characterizing the dispersion of energy, internal energy, chemical affinity, the thermodynamic flow (TFL), the thermodynamic force (TFO) and the rate of entropy production (diS/dt) which is the product of TFL and TFO [58]. Open living contractile systems exchange energy and matter with the environment. In statistical mechanics, the canonical complex can be applied to complex open systems such as contractile muscle tissues. Combining statistical mechanics and the A. Huxley formalism provides a powerful tool to show the link between the chemo-mechanical properties of molecular motors CB and thermodynamic characteristics. This has led us to observe that striated and smooth muscles behave near equilibrium. This means that the chemical affinity is ≪ RT (R: gas constant, T: Kelvin temperature). Moreover, open contractile systems behave in a linear regime. This means that TFL varies linearly with TFO. In the linear regime, in which the phenomenological Onsager laws are observed [59, 60], an open system can approach a linear regime [58]. Thus, the entropy production rate diS/dt can achieve a minimum level. This characterizes the stability criterion of a stationary regime. The irreversibility of chemical processes can be quantified by diS/dt. First, we determine the CB molecular properties and the probability of the different steps of the CB cycle by using the A.F. Huxley equations. Secondly, we can apply statistical mechanics to various muscles and establish that muscles behave near equilibrium and in a stationary linear regime [57, 59].

Besides muscular systems, other tissues also present contractile behavior. In a similar manner to that observed in muscle tissues, placental stem villi can contract after potassium chloride stimulation or after application of an electric field [61, 62]. We have found that the Frank-Starling phenomenon is also present in stem villi of human placenta [46]. The ultrastructure of human placental stem villi seems to be relatively highly organized and this suggests a possible sliding of actin filaments along myosin filaments as reported in both striated and smooth muscles. The hyperbolic tension-velocity relationship is also observed in human placental stem villi [45]. This makes it possible to determine their molecular characteristics and thermodynamic properties [48]. The fundamental difference between the muscles and placenta is the dramatically low shortening velocity of the placenta compared to that of muscles. This means that placental stem villi develop a very low tension compared to that of muscles. In addition, the kinetics of NMMIIA and the NMMIIA maximum ATPase activity are much lower in human placenta than those observed in muscles [57]. Importantly, the unitary forces generated by one CB interaction and the thermodynamic CB efficiency are of the same order of magnitude in both muscles and non-contractile stem villi of the placenta.

We have recently shown that mesenchymal stem cells (MSCs) from human bone marrow collected after a femoral neck fracture present the same mechanical properties as those of human placental stem villi when MSCs are included in collagen scaffolds in the presence of human platelet lysate (HPL) TGF-β [63]. Under these conditions, MSCs differentiate into myofibroblasts. The TGF-β induced canonical WNT/β-catenin pathway increases mesenchymal stem cell (MSC) differentiation and fibroblast differentiation into myofibroblasts.

Myofibroblasts play a key role in tissue repair processes such as scarring of the skin. Canonical WNT pathways and TGF-β promote tissue fibrosis in the heart, lungs, liver and kidneys. Finally, myofibroblasts also play a key role in several cancers [64].

Within myofibroblasts, four systems play an important key role in the initiation of the fibrotic process. These systems are PPARγ, canonical WNT/β-catenin signaling, TGF-β and the Hippo pathway. These pathways operate in the pathogenesis of fibrotic processes. The pathways share the molecular mechanisms regulating the cytosolic/nuclear transfer of their transcriptional activators.

## PPARγ

PPARγ is a pleiotropic transcription factor that belongs to the superfamily of nuclear hormone receptors [65]. PPARγ heterodimerizes with the retinoid X receptor to bind PPAR response elements (PPRE) [66]. PPARγ is stimulated by ligands, which couple coactivators (p300/CBP and P160). PPARγ is observed in many cells including adipose tissue, muscles, brain and cells of the immunity system. PPARγ stimulates the expression of numerous target genes and thus, regulates glucose homeostasis, insulin sensitivity, lipid metabolism, the immune response and inflammation [67]. PPARγ is also present in fibroblasts [68]. Two isoforms of PPARγ have also been observed. PPARγ1 is expressed in macrophages, epithelial cells, endothelial cells and vascular smooth muscle cells, while PPARγ2 is observed in adipose tissue, where it regulates the adipogenesis process.

PPARγ is stimulated by natural agents such as prostaglandin J215d (15d-PGJ2), lysophosphatidic acid and nitrolinoleic acid. PPARγ is stimulated by synthetic ligands, such as thiazolidinediones (TZDs) and acid derivatives, including triterpenoids oleanic (2-cyano-3,12-dioxoolean-1,9-dien-28-oic acid (CDDO)). TZDs enhance insulin sensitivity in several tissues and stimulate glucose tolerance and insulin sensitivity in patients with type 2 diabetes [69]. TZDs operate on the initiators of the glucose transporter (GLUT-2) and glucokinase (GK). Moreover, PPARγ participates in the regulation of connective tissue, activation of mesenchymal cells, differentiation, and cell survival by linking with the metabolism and fibrogenesis [14]. PPARγ abnormalities are present in many pathophysiological conditions, including obesity, cancer, diabetes, atherosclerosis and multiple sclerosis. Many TZDs have been used in the treatment of diabetes mellitus. Nevertheless, side effects induced by TZDs have also been reported [70]. Benefits enhanced by TZDs are mitigated by the possibility for water retention, congestive heart failure, weight gain, bone loss and liver kidney diseases. It therefore seems to be of the utmost importance to initiate the development of new molecules to diminish the negative effects induced by TZD use. PPARγ also plays a major role in controlling cardiovascular rhythms by monitoring changes in the circadian rhythms of blood pressure and heart rate via Bmal1 [71].

PPARγ has a vital role in the fibrosis process and in cutaneous lesions. When the skin is disturbed due to injury, resident fibroblasts are submitted to mechanical stress. This stress, coupled with a TGF-β1 release from immune cells and platelets at the site of the wound, leads to the migration of dermal fibroblasts from normal surrounding skin near the site of injury [72]. Thus, fibroblasts differentiate into myofibroblasts. PPARγ play a major role in decreasing the fibrotic process by antagonizing TGF-β1. PPARγ agonists decrease the deposition of TGF-β induced collagen and myofibroblast differentiation [73]. Excessive scarring and/or chronic wounds may constitute a major problem during tissue injury. The discovery of new drugs to minimize fibro-proliferative processes is of considerable significance. PPARγ could be useful in the prevention of excessive scarring. It is important now to clarify whether the PPARγ agonists induce positive effects on the repair of skin tissue in humans.

## PPARγ modulators

PPARγ expression is decreased by many cytokines, chemokines, and intracellular pathways decrease, such as TFG- β1, canonical WNT/β-catenin pathway, TNF-α, Interleukin (HE)-1β, IFN-γ, HE-13, connective tissue growth factor (CTGF), leptin and lysophosphatidic acid (LPA) [74, 75]. The transcription factor COUP II is a canonical WNT target which reduces PPARγ. Hypoxia reduces PPARγ expression [76]. Other molecules stimulate PPARγ expression, such as adiponectin, TZDs, L-carnitine, statins, eplerenone, and irbesartan [77]. Adiponectin helps stimulate PPARγ2 expression and reduce activation and production of IL-6 NF-kappaB induced by LPS in adipocytes [78]. Other transcription factors modulate PPARγ in a positive manner, such as C/EBP, ELF proteins, and NF-E2-related factor 2 (Nrf2), the receiver of the bile acid receptor farnesoid X (FXR), which binds to the canonical WNT pathway [79].

### The canonical WNT/β-catenin pathway

**Fig. 2:**
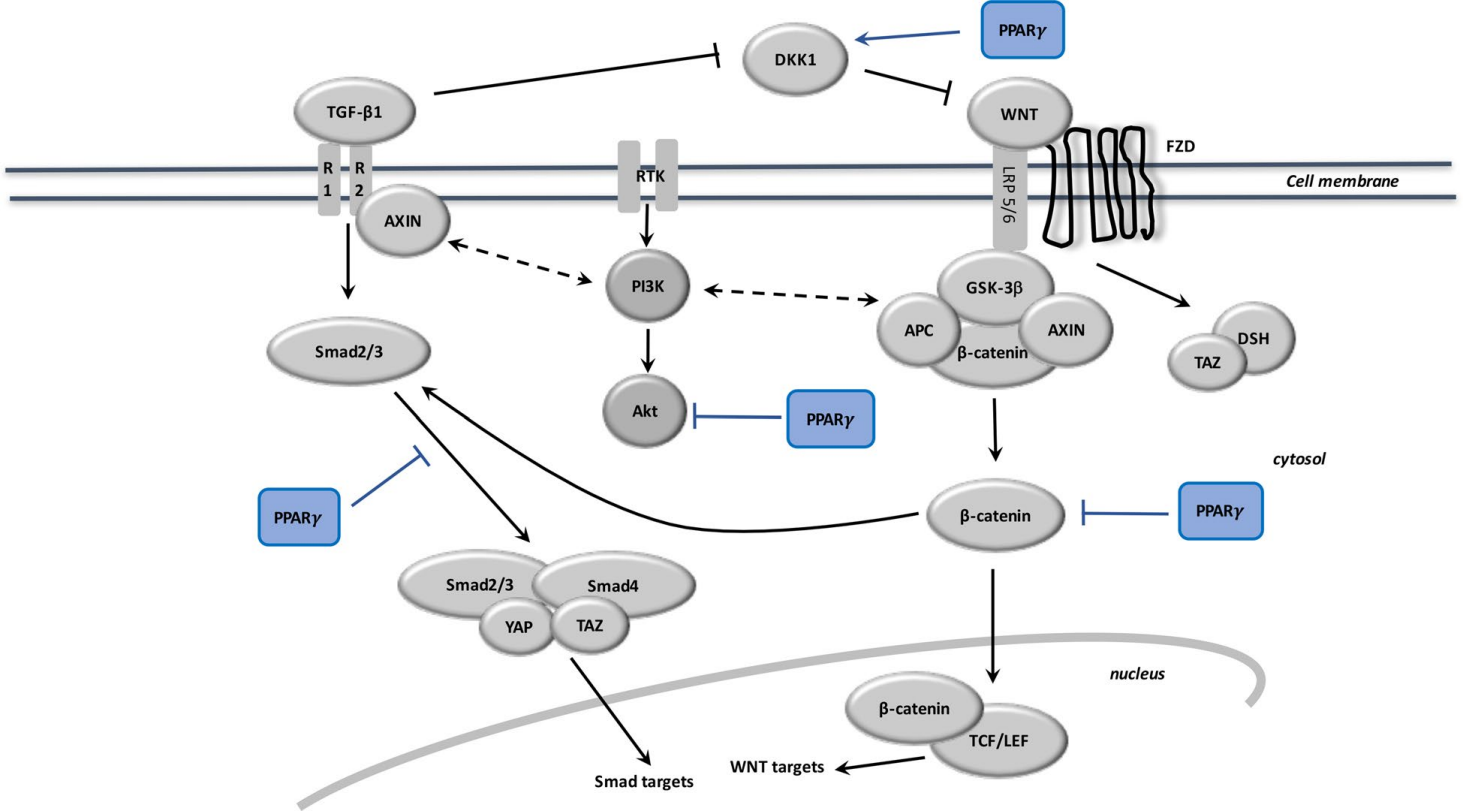
TGF-β1 effects on the balance between the canonical WNT/β-catenin pathway and PPARγ. WNT activation inhibits the β-catenin destruction complex, which results in the β-catenin accumulation in the cytosol and then its translocation to the nucleus for activating WNT target genes. Following WNT stimulation, TAZ inhibits the phosphorylation of DSH and dissociates it from the β-catenin destruction complex. The destruction complex is inhibited because YAP and TAZ dissociate from the complex. Following TGF-β stimulation, AXIN promotes the tail-phosphorylation of Smad2/3. The activated Smad2/3-Smad4 complex associates with TAZ and YAP and then translocates to the nucleus for activation of Smad targets. TGF-β1 induces Smad2/3 and PI3K/AKT pathway activation. PPARγ inhibits β-catenin/TCF-LEF-induced activation of the WNT target genes. TGF-β also enhances WNT signaling through inhibition of DKK1. PPARγ actives DKK1 and inactivates PI3K/AKT. APC adenomatous polyposis, DKK1 Dickkopf-1, DSH disheveled, FZD frizzled, GSK-3β glycogen synthase kinase-3β, LRP5/6 protein 5/6 connected to the low-density lipoprotein receptor, PPARγ peroxisome proliferator-activated receptor gamma, TCF/LEF T cell factor/lymphoid enhancer factor, *TGF* transforming growth factor

The canonical WNT/β-catenin pathway has a major role in metabolism, embryonic development, cell fate, and the epithelial-mesenchymal transition (EMT) [80]. Stimulation of canonical WNT signaling leads to an increase in the nuclear and cytosolic levels of β-catenin (Fig. 2). In the “on” state, canonical WNT ligands interact with both the canonical WNT receptor frizzled (FZL) and the related protein 5/6 LDL receptor (LRP5/6), FDZ complex, and with disheveled (DSH). This dysregulates the destruction complex to prevent β-catenin degradation in the proteasome. The destruction complex is composed of a suppressor, adenomatous polyposis coli tumor (APC), AXIN and glycogen synthase kinase-3β (GSK-3β). β-catenin translocates to the nucleus to bind the T cell/lymphoid amplifier (TCF/LEF) transcription factors. This phenomenon stimulates several β-catenin target genes (c-Myc, Cyclin D, Cox 2, AXIN, PDK, MTC-1) [81, 82]. In the absence of WNT ligands, the destruction complex phosphorylates β-catenin, which is destroyed in the proteasome. Dysregulated canonical WNT signaling has been observed in numerous diseases such as cancers [4, 83–85]. Stimulation of the WNT/β-catenin has been observed in the liver, skin, lungs, kidneys and heart in fibrotic processes [86, 87].

## Interplay between the WNT pathway and PPARγ

Canonical WNT signaling is negatively regulated by PPARγ ligands [84, 88, 89]. Stimulation of the canonical WNT/-β-catenin pathway is a major phenomenon involved in the fibrotic pathogenesis [90]. TZDs stimulate DKK1, which is an inhibitor of the canonical WNT pathway (Fig. 2), and block the differentiation of fibroblasts [91]. GW11929, a non-TZD PPARγ agonist, decreases the transcription of β-catenin [92]. The inhibitory role induced by canonical WNT signaling on PPARγ has been observed to be the phenomenon that leads to the anti-adipogenic effects [93]. During osteoblastogenesis, WNT signaling is directly activated by the inhibition of both PPARγ and the enhancer-binding protein CCAAT/ [94]. Thus, stimulation of WNT/β-catenin signaling and downregulation of GSK-3β activity leads to the activation of fibroblast differentiation and fibrotic processes [95].

In addition, downregulation of PPARγ enhanced by WNT ligands can be carried by non-canonical pathways [93]. The non-canonical WNT pathway through CaMKII-TAK1-NLK-TAB 2 inhibits the transactivation of PPARγ.

## TGF-β1

TGF-β are composed of three similar structural proteins, namely TGF-β1, TGF-β2 and TGF-β3. TGF-β receptors are transmembrane proteins and include the type I receptor (TβRI) and type II receptor (TβRII) (Fig. 2). TGF-β1 can bind TβR2 but not TβR1. TGF-β1 is secreted and deposited in ECM as a large latent complex, comprising a latent TGF-β1 binding protein bound to a small latent complex. Integrins α_v_β5 and α_v_β6 stimulate TGF-β1. In addition, TGF-β1 stimulates Smad signaling and non-Smad signaling, including MAPK, Rho, and PI3K-AKT. TGF-β1stimulates PI3K/AKT by activating focal adhesion kinase (FAK) [96, 97]. FAK is a non-receptor protein tyrosine kinase that is phosphorylated in response to integrin clustering and growth factor-mediated migration [98]. FAK is recruited to focal adhesion following integrin clustering [99], and is subsequently activated by phosphorylation at Tyr297. Activation of the phosphorylation of FAK is correlated with its increased catalytic activity [100, 101] and is required for the recruitment of p85, a regulatory subunit of PIEK/AKT [102]. Thus, FAK is involved in myofibroblast differentiation via TGF-β1 [103]. FAK is involved as an upstream activator of AKT and then contributes to fibrogenesis [104, 105]. Several fibrotic disorders present an activation of the TGF-β1 pathway. Thus, TGF-β1 is elevated in glomerular and tubulo-interstitial diseases, in diabetes mellitus, in lungs, in the broncho-alveolar lavage of patients with SSc, and hypertrophic and restrictive cardiomyopathy [106–108].

## Interplay between PPARγ, canonical WNT and TGF-β1 (Figs. 2 and 3)

The observed link between TGF-β1, canonical WNT/β-catenin and PPARγ has been well documented [77]. TGF-β1 can activate canonical WNT signaling, and can decrease PPARγ expression. In contrast, PPARγ decreases the TGF-β1/WNT/β-catenin pathway. PPARγ ligands lead to a decrease in TGF-β1 through PI3K/AKT signaling [109]. TGF-β1 is a major controller of fibrosis and an interesting target in fibrosis [110]. TGF-β1 leads to fibroblast differentiation into myofibroblasts in the human lung. The fibrosis process is decreased through the inhibition of TGF-β1 by means of PPARγ agonists [111]. PPARγ induces protection against excessive fibrogenesis [112]. In the eye, PPARγ ligands (15-deoxy-prostaglandin J2 delta 12,14, troglitazone, rosiglitazone) may remove corneal myofibroblasts [113].

**Fig. 3:**
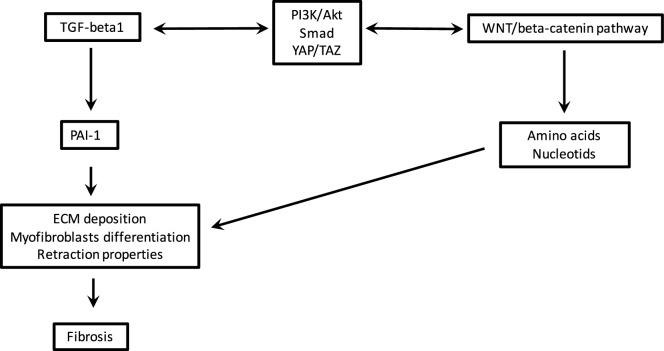
Schematic representation of the fibrosis process with the interaction between TGF-β1 and the canonical WNT/β-catenin pathway

The opposing interplay between PPARγ and TGF-β1 would in part support the fibrosis process. TGF-β1 activation promotes the differentiation of fibroblasts into myofibroblasts and negatively regulates PPARγ expression. TGF-β1 reduces PPARγ expression in both fibroblasts [114] and hepatic stellate cells [115]. In contrast, PPARγ agonists directly disrupt the TGF-β pathway and its synthesis [116]. PPARγ ligands (15d-PGJ2 and troglitazone) inhibit the expression and synthesis of collagen in fibroblasts enhanced by TGF-β1 [116, 117]. Troglitazone, 15d-PGJ 2 and CDDO prevent α-SMA expression [118]. PPARγ agonists decrease TGF-β1-induced CTGF expression [119]. In pulmonary fibrosis induced by bleomycin, the diminution of canonical WNT/TGF-β1 signaling decreases LRP5 and reduces fibrosis [120]. TGF-β1 also enhances WNT signaling by inhibiting Dickkopf-1 (DKK1) [121]. Even if DDK1 inhibits TGF-β-induced fibrosis, the decrease in DKK1 activity leads to the stability and nuclear accumulation of β-catenin in epithelial cells and fibroblasts, which encourages the fibrotic process.

## The Smad pathway

The Smad pathway helps us to better understand the association between TGF-β1, the WNT pathway and PPARγ (Fig. 2). In myofibroblasts, stimulation of the canonical Smad pathway controls the intracellular TGF-β1 pathway. TGF-β1 binds TGFβR2 to recruit TGFβR1. This mechanism helps induce the formation of a heterotetramer and phosphorylation of Smad 2 and Smad 3, which then binds Smad 4 (Fig. 2). This activates the recruitment of coactivators, including p300 histone acetyltransferase, and targets gene transcription [122].

Non-Smad pathways exist as p38 MAP kinase and JNK, TGF-β stimulated kinase (TAK1), the focal adhesion kinase and PI3K-AKT [120]. Furthermore, CTGF, the platelet-derived growth factor (PDGF), IL-4, IL-6, IL-8 and IL-13 interact with TGF-β1 and participate in the fibrotic process [123]. PPARγ ligands disrupt both Smad-dependent and Smad-independent TGF-β1 signaling. Downregulation of PPARγ promotes the canonical Smad signaling 2/3. In humans, the PPARγ promoter has two Smad connecting elements [115].

## Interplay between Smad/TGFβ1 and canonical WNT/PPARγ pathways in fibrosis

Regulation of the homeostasis of connective tissue represents a recently discovered role of PPARγ, especially in tissue repair and fibrotic processes. In general, an opposing interplay has been observed between PPARγ expression and the fibrotic process. In human fibrosis diseases, PPARγ expression is decreased, as in lung, scarring, alopecia [124], liver [125], and kidney [126] diseases. In numerous fibrotic processes, decreased PPARγ expression and/or activity precedes the fibrotic process, suggesting a causal role for fibrosis [5]. In mice with systemic sclerosis, PPARγ expression is reduced in the skin tissue and rosiglitazone abrogates bleomycin-induced scleroderma and blocks responses by a PPARγ profibrotic mechanism [116]. PPARγ-deficient fibroblasts show increased TGF-β1, collagen type 1, and α-SMA [127].

The Smad pathway could explain the anti-fibrotic role of PPARγ ligands. PPARγ, through the inhibition of TGF-β signaling, can help control fibrosis processes. Thus, overexpression of PPARγ participates in the regulation of fibrosis development in skin, lung, pancreas, heart and liver [114]. PPARγ agonists inhibit TGF-β1/Smad3 signaling in human hepatic stellate cells [128]. Activated PPARγ ligands suppress the production of collagen in accordance with Smad targeting of the transcription coactivator p300 [117]. Triterpenoid improves fibrosis [118]. In fibroblasts, CDDO can prevent TGFβ1-induced fibrotic processes by removing the Smad transcription and the downregulation of the AKT pathway [118]. Troglitazone, ciglitazone and 15d-PGJ 2 results in an overexpression of the hepatocyte growth factor (HGF), which leads to activation of the TG-interaction factor (TGIF), a co-Smad transcriptional repressor, and prevents the TGF-β1-induced fibrotic process [129]. The differentiation of human peripheral blood fibrocytes is mediated by TGF-β1and PPARγ [130]. Then, troglitazone inhibits the TGF-β1-induced SAPK/JNK signaling that reduces the Smad2 activity and modifies myofibroblast differentiation. PPARγ ligands diminish TGF-β-mediated Erg1 signaling [131].

PPARγ can exert a negative control on the synthesis of the collagen-induced profibrotic signal and blunting fibrosis in several pathological conditions [5, 6]. Moreover, in lung and cultured skin fibroblasts, hepatic stellate cells and mesangial cells, PPARγ ligands (15d-PGJ2 and rosiglitazone) decrease the differentiation of fibroblasts into myofibroblast, the synthesis of collagen, fibronectin and TGF-β1 [125, 132]. PPARγ ligands can stop the epithelial-mesenchymal transition of alveoar epithelial cells induced by TGF-β1 [133].

In mice, adiponectin prevents fibrosis in the liver [134]. In numerous animal models of fibrosis, PPARγ ligands decrease fibrosis in numerous organs, including heart [135], lung [136], liver [137] and kidney [138]. PPARγ 15d-PGJ2 agonist and rosiglitazone decrease pulmonary fibrosis induced by systemic sclerosis [139].

PPARγ-induced tensin homologue, PTEN, induces an anti-fibrotic effect in pulmonary fibrosis and SSc [140]. PTEN inhibits the production of collagen and myofibroblast differentiation [141]. PPARγ suppresses TGF-β1-enhanced EMT in alveolar epithelial cells and metastasis, without blocking the Smad pathway [142]. In PPARγ deficient mice, phosphorylation of Smad3 is increased and α-SMA and type 1 collagen expression is stimulated [127]. WNT3a promotes myofibroblast differentiation through the upregulation of TGF-β1. This occurs in a Smad2 β-catenin dependent manner [143]. In addition, it has been observed that aerobic glycolysis is enhanced by TGF-β1 activation [144].

## Non-Smad-dependent signaling and fibrosis

PPARγ agonists may prevent TGF-β1-induced fibrosis, regardless of the Smad pathway [73, 116]. Thus, rosiglitazone does not inhibit Smad2 phosphorylation. In lung fibroblasts, myofibroblastic dedifferentiation by PTEN diminishes collagen expression and α-SMA [140]. PTEN downregulates the PI3-OH kinase, (PI3K)-AKT. PI3K leads to the formation of phosphatidylinositol 3,4,5-triphosphate (PIP3) from PIP2. AKT is stimulated by PIP3. PTEN is a PIP3 phosphatase that acts in a way that opposes the role of PIK3. PPARγ ligands suppress TGF-β1 and target the PI3K/AKT [109], which induces the differentiation of myofibroblasts.

## The Hippo pathway

Yes-associated protein 1/transcription coactivator and PDZ-binding motif (YAP/TAZ) are transcription co-activators of the Hippo kinase complex [145]. Hippo signaling controls organ size and the regeneration and self-renewal of cells. Hippo signaling consists of numerous components including serine/threonine protein kinases (MST1/2), MOB kinase activator 1 (MOB1), Salvador (SAV) and the serine/threonine protein kinase-(LATS1/2). When the kinase Hippo is in the “on” state, YAP and TAZ are phosphorylated and lead to a phosphodegron. YAP and TAZ may be sequestered in the cytosol or degraded by β-TrCP proteins. Activated Hippo signaling induces phosphorylation of YAP and leads to a reduction in the β-catenin level.

During the fibrosis process, F-actin polymerization downregulates MST1/2 activity, which inactivates the Hippo complex. Then, YAP and TAZ are released and translocated to the nucleus where they bind with several transcription factors, including transcription factor Runt (RunX) and the TEA domain family member (TAED), to activate gene transcription.

YAP and TAZ are involved in the activation of myofibroblasts and the initiation of fibrosis. In biopsies of idiopathic pulmonary fibrosis, both YAP and TAZ levels are high, and induce activation of fibroblasts and then fibrosis. In lung fibroblasts and mouse liver, knockdown YAP and TAZ reduce procollagen, α-SMA, and the inhibitor of plasminogen activator 1, which have all been linked with myofibroblast differentiation [146].

## Interaction of TGF-β pathways, WNT, Smad and the Hippo pathway in fibrosis (Fig. 4)

An intricate interplay exists in myofibroblast differentiation. During the healing of skin wounds in mice, observations have shown that both YAP and TAZ are increased. TGF-β1 is stimulated in skin wound healing. This suggests a link between YAP and TAZ stimulation and TGF-β1 activation [147]. YAP and TAZ also control the activity and expression of the TGF-β1 component pathway including Smad-2. WNT3a induces myofibroblast differentiation by improving TGF-β1 through phosphorylation of Smad2 in a dependent β-catenin manner [143]. An interplay has also been observed between the YAP/TAZ pathway and TGF-β. In epithelial cells, TAZ interacts with Smad2/Smad3 [148]. TAZ binds with Smad2/3 to increase the transfer of Smad2/3 in the nucleus and thus the transcription of PAI-1 and Smad7 target genes. Furthermore, in mesothelioma cells, YAP binds with Smad3 [149]. In the cytosol, TAZ is linked with canonical WNT signaling by an interaction of TAZ-β-catenin [150]. During the “on”-state of WNT signaling, the release of β-catenin from the destruction complex alters TAZ degradation, leading to the cytosolic accumulation of both β-catenin and TAZ. This impact of TAZ on the WNT pathway is independent of its function as a mediator of Hippo signaling. In the “off”-state of the WNT pathway, cytosolic YAP/TAZ specifically binds with AXIN [151].

**Fig. 4:**
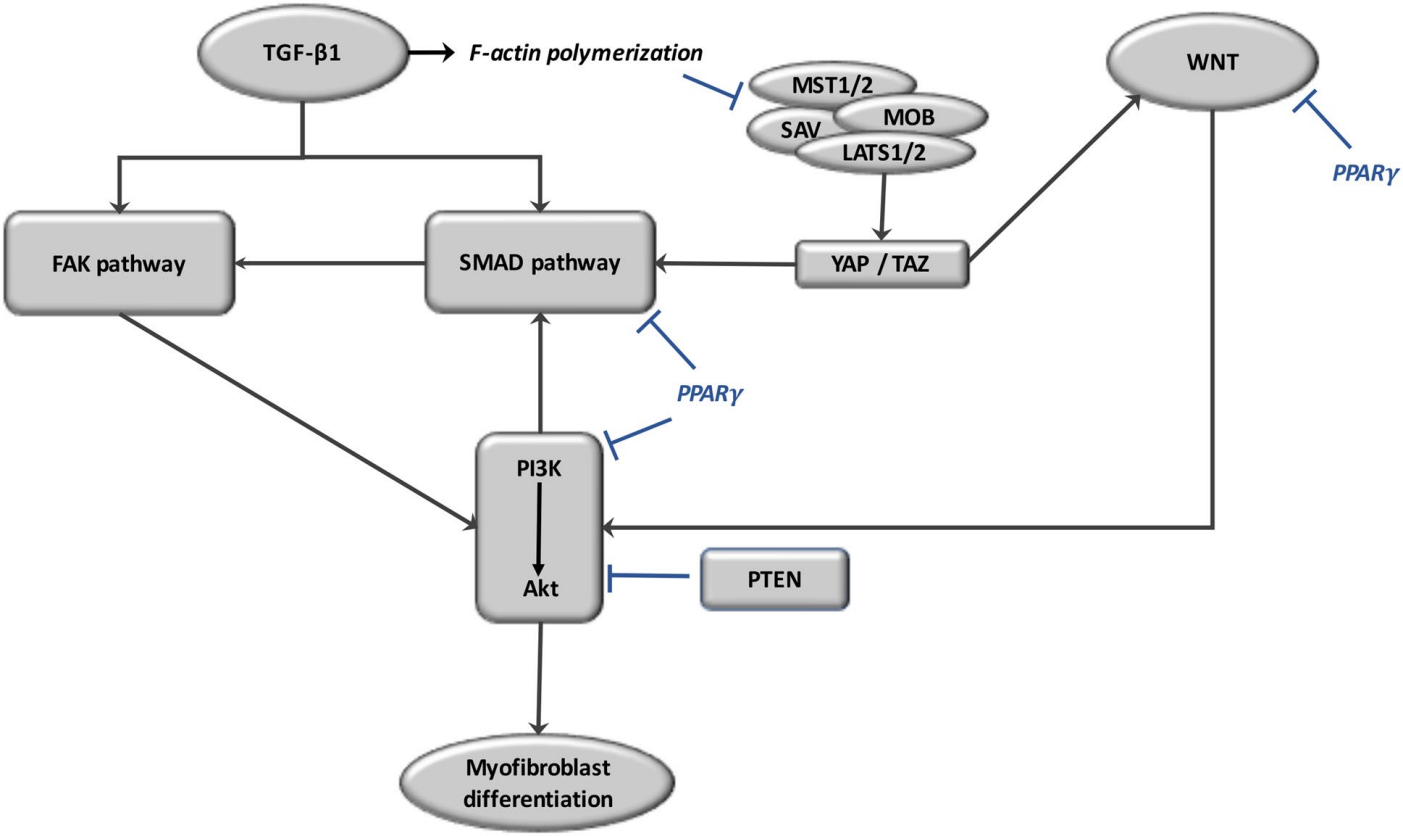
Interactions of TGF-β pathways, WNT, Smad and the Hippo pathway in fibrosis. TGF-β and WNT induce myofibroblast differentiation by activating Smad, FAK and then the PI3K/AKT pathway. TGF-beta stimulates F-actin polymerization, which inhibits the signaling MST1/2, MOB, SAV, and LATS1/2 leading to YAP/TAZ complex with Smad pathway to translate to the nucleus. PPARγ ligands inhibit the TGF-β-induced PI3K/AKT, beta-catenin accumulation and, in part through the targeting of FAK, the activation of AKT. YAP/TAZ yes-associated protein 1/transcription coactivator and PDZ-binding motif, MST1/2 serine/threonine protein kinases, MOB1 MOB kinase activator 1, SAV Salvador, LATS1/2 serine/threonine protein kinase

## New molecular insights into fibrosis

Several potential cellular sources of extracellular matrix-producing cells can promote fibrosis [152]. Mesothelial cells derive from the embryonic mesoderm cell layer and play a protective role in the internal organs of the body by producing a lubricating fluid that helps protect the body against infection. TGF-β has been shown to induce a reformation of the “mesothelial-to-mesenchymal transition” in differentiated human mesothelial cells, via a positive α-SMA loop [153]. Endothelial cells can differentiate into mesenchymal cells, by an “endothelial-to-mesenchymal-transition”, and then contribute to fibrosis [154]. Vascular smooth muscle cells typically express α-SMA and induce collage type I production via autocrine activation of TGF-β1 and involvement of mitogen-activated protein kinase pathways [155]. TGF-β is considered as one of the common master switches for induction of the fibrotic program during chronic phases of inflammatory diseases in many organs. Other important soluble pro-fibrogenic mediators include the connective tissue growth factor (CTGF) and members of the platelet-derived growth factor (PDGF) family of growth factors. While CTGF is thought to bind with TGF-β and enhance its binding to membrane-bound receptors, members of the PDGF family are potent mitogens and chemoattractant for fibrogenic cells in most organs driving the recruitment and proliferation of these cells at sites of tissue injury [156]. In parallel, several interleukins, such as IL-1 and IL-6, are critical proinflammatory contributors to the pathogenesis of fibrosis [153]. Lipid laden macrophages (i.e. foam cells) release oxidized phosphatidylcholine to induce the production of TGF-β and then the promotion of M2 polarization of macrophages during the fibrosis process (Kubo 2018). Recent targets in fibrosis biomarker research include circulating micro-RNA (miRNA), long non-coding RNA, epigenetic changes, mitochondrial DNA or microbiome signatures in the stool [157–159]. Many new directions of research are therefore aimed at improving strategies for targeting drugs at myofibroblasts [160]. Potential targeting systems include modified albumin, hydrogel, peptide, nanoparticle, aptamer and antibody-based systems, which demonstrated promising myofibroblast targeting capacities in preclinical models [160–162].

## Conclusion

Several signaling systems can exercise control over the differentiation of fibroblasts into myofibroblasts. Furthermore, myofibroblasts have a major function in cell fibrosis in several organs, such as the kidneys, heart, lungs, and liver, and in wound healing. Canonical WNT/β-catenin signaling and PPARγ behave in an opposing manner, and thus participate in respectively promoting and reducing fibrosis. TGF-β has a major role in the fibrotic process by acting on the contractile properties of myofibroblasts (Fig. 3). TGF-β regulates the differentiation of fibroblasts into myofibroblasts and can act as a driver by upregulating the canonical WNT pathway and downregulating PPARγ.

With respect to the human clinical approach, no treatment has yet been able to stop and regress fibrosis. Many attempts have used antagonist antibodies or small molecules that act on the TGF-β, canonical WNT, Smad and YAP/TAZ cascades. Although considerable therapeutic efficacy has been observed in animal models [163], evidence from human trials to date is insufficient. Indeed, some studies have indicated that there may be serious side effects, while other human trials have been more encouraging, especially in scleroderma [164]. in the lack of sufficient results to ensure effective control of the fibrotic process may be partly due to the complex relationship between the different signaling systems (TGF-β, canonical WNT/β-catenin, and Smad-Hippo/YAP/TAZ), which may be partially opposed to one another. PKC-δ and angiotensin inhibitors also exhibit remarkable anti-profibrotic roles and can be used to in implementing efficient therapies in the fibrotic process [110]. In addition, PPAR γ may interrupt the actions of profibrotic TGF-β, myofibroblast differentiation and excess collagen production.

## Data Availability

Not applicable.

## Abbreviations

APC: adenomatous polyposis
DKK1: Dickkopf-1
DSH: disheveled
FZD: frizzled
GSK-3β: glycogen synthase kinase-3β
LRP5/6: protein 5/6 connected to the low-density lipoprotein receptor
PPARγ: peroxisome proliferator-activated receptor gamma
TCF/LEF: T cell factor/lymphoid enhancer factor
TGF: transforming growth factor
YAP/TAZ: yes-associated protein 1/transcription coactivator and PDZ-binding motif
MST1/2: serine/threonine protein kinases
MOB1: MOB kinase activator 1
SAV: Salvador
LATS1/2: serine/threonine protein kinase
